# Supplementary material for: Large scale and integrated platform for digital mass culture of anchorage dependent cells
Source: Nat Commun. 2019 Oct 23;10:4824. doi: 10.1038/s41467-019-12777-3 (PMC6811641; doi:10.1038/s41467-019-12777-3)
Supplement: Supplementary file 1 — Supplementary Information [file 41467_2019_12777_MOESM1_ESM.pdf]

Supplementary Information

Large Scale and Integrated Platform for Digital Mass Culture of Anchorage Dependent Cells

Cho et al.

The PDF file includes:

Supplementary Note 1, Supplementary Figures 1-24, Supplementary Table 1, and Supplementary References.

1. Supplementary Note 1

1.1. FEM and analytical modeling of dissolved oxygen diffusion in multilayer engineered substrates

Cells adherent on culture substrates in cell culture experience a lower concentration of dissolved oxygen as the depth of the culture medium increases[1].To elucidate how perforations on the cell platform help oxygen diffuse, finite element modeling (FEM) was conducted using the commercial package COMSOL/MultiPhysics 5.2. The time-dependent oxygen diffusion process in the culture media is governed by Fick's law,

$$(1) \qquad \frac{\partial c}{\partial t} = D(\frac{\partial^2 c}{\partial x^2} + \frac{\partial^2 c}{\partial y^2} + \frac{\partial^2 c}{\partial z^2})$$

where c is the concentration of the oxygen, $D \approx 2.6 \times 10^{-9}\, m^2/s$ is the constant diffusion coefficient of Oxygen[2,3], x, y, z are spacial coordinates. The investigated culture media domain (glucose environment in reality) is $60\, mm \times 60\, mm \times 13\, mm$ with coordinate origin located at the center on the bottom surface as shown in fig. S6b. Because the top surface (z=13 mm) is contacting the ambient environment, oxygen concentration on that surface is assumed to be the saturation concentration, which approximately equals $c_0 \approx 2.18 \times 10^{-4} mol/L$ at temperature ~35 ℃[4]. Due to their contact wit2h the container, oxygen flux through four vertical side surfaces ($x, y = \pm 30\, mm$) and the bottom surface (z=0) is zero. Cells on the platform consume oxygen and this leads to an oxygen flux rate $f = -1.73 \times 10^{-4}\, mol/(m^2 \cdot h)$ for each layer (the negative sign means oxygen dissipation)[5]. Fig. S6b shows normalized oxygen concentration c/c_0 for the flat and engineered substrates at 12 hr and 24 hr. The results clearly suggest that perforations enhance the diffusion of oxygen, which leads to accelerated cell growth.

1.2 Detailed descriptions on measuring circuits

Both impedance measurement and electrical stimulation share same circuits (Fig. S11). Only difference is that a switching chipset (U7, Fig. S10a and S12) relays the signal generated from DAC0 to a LPF (U4, Fig. S10a and S12) input in case of impedance measurement, whereas DAC0 is directly connected to the input of a multiplexer (U3, Fig. S11a and S12) in case of electrical stimulation. The LPF smoothens the digitized signals generated from DAC0 to inject sinusoidal signals to cells in case of impedance measurement. The multiplexer receives digital signals from Arduino DUE's digital outputs (D35–39, Fig. S11a) to decide certain interconnection configuration. A transimpedance amplifier (U1A, Fig. S11a and S12) and an inverter (U2B, Fig. S11a and S12) are utilized for converting electrical current to voltage and amplifying it. The inverter is necessary because the transimpdance amplifier inverts the input signals. Because Arduino DUE's ADC only accepts positive voltage as measurable inputs, DAC1 and a resistor (R1, Fig. S11a) of appropriate resistance value supply offset currents. Temperature sensing utilizes a typical voltage dividing circuit. A digital output (D44, Fig. S11a) applies digital signal to the temperature sensor and an analog input (A1) measures corresponding voltage between ground and a load resistor (R20, Fig. S11a). Because ion sensors (pH/K^+ sensors) typically have high impedance, voltage followers (U9, U13; Fig. S11a and S12) are located between the sensors and ADCs. Output impedance of the voltage followers are low enough so that the exact open circuit voltage measured by the sensors can be transferred to the ADCs. Each heater in the heater array requires different voltage to reach the same temperature because each heater has different resistance of interconnections. The programmable voltage regulators (U10, U11; Fig. S11a and S12) can provide regulated voltages by selecting appropriate capacitor and resistors. A switching chip (U12, Fig. S11a and S12) selects the desired heaters by using digital signal delivered from Arduino DUE.

Supplementary Figure

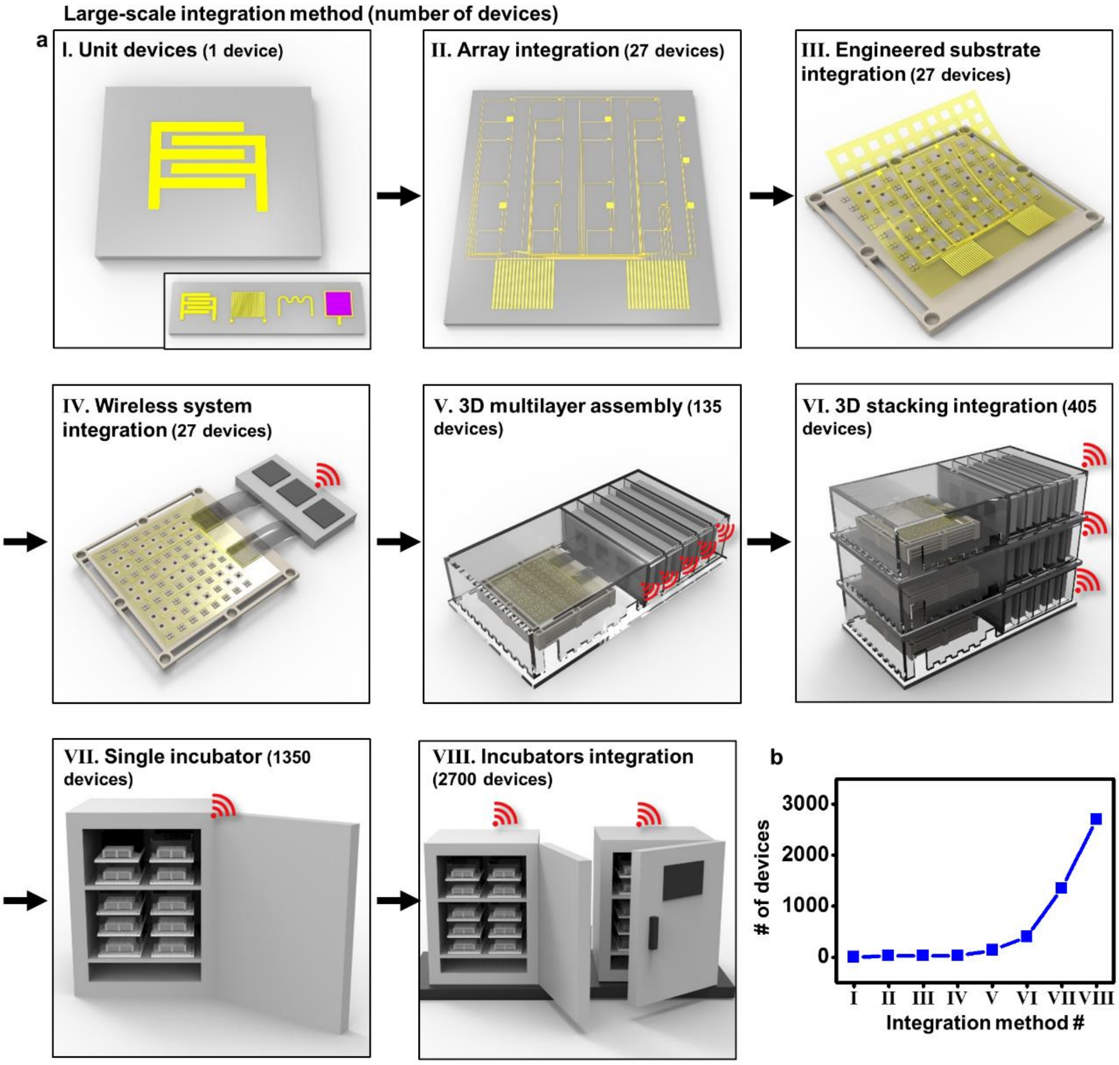

Supplementary Figure 1. Integration methods of large-scale, integrated, and smart cell culture platform. **a**, Schematic illustrations of each step of the integration methods. The unit devices such as impedance, temperature, pH, and K^+ sensors, and electrical/thermal stimulators are integrated into 3D arrays to achieve the large-scale cell culture. **b**, Plots showing the exponential increases of the number of devices

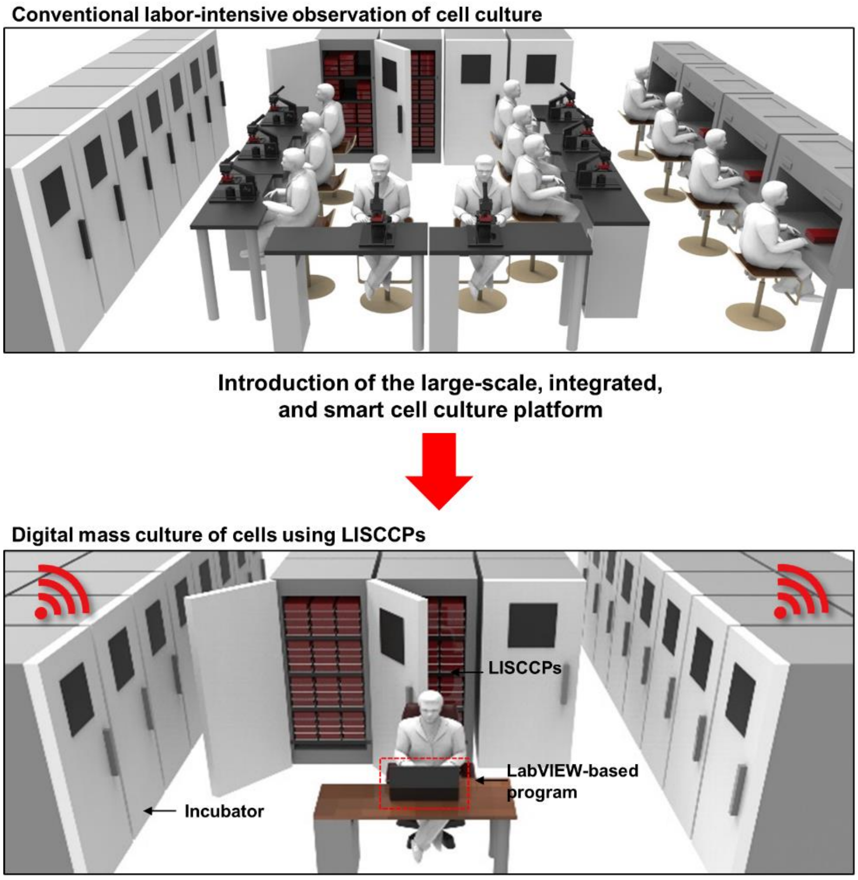

Supplementary Figure 2. Concept illustration of the transformation of the cell culture factory. The conventional cell culture system where high manpower and high labor are required can be transformed into the digital mass culture of anchorage-dependent cells in which a single person can monitor and control the large-scale cell culture.

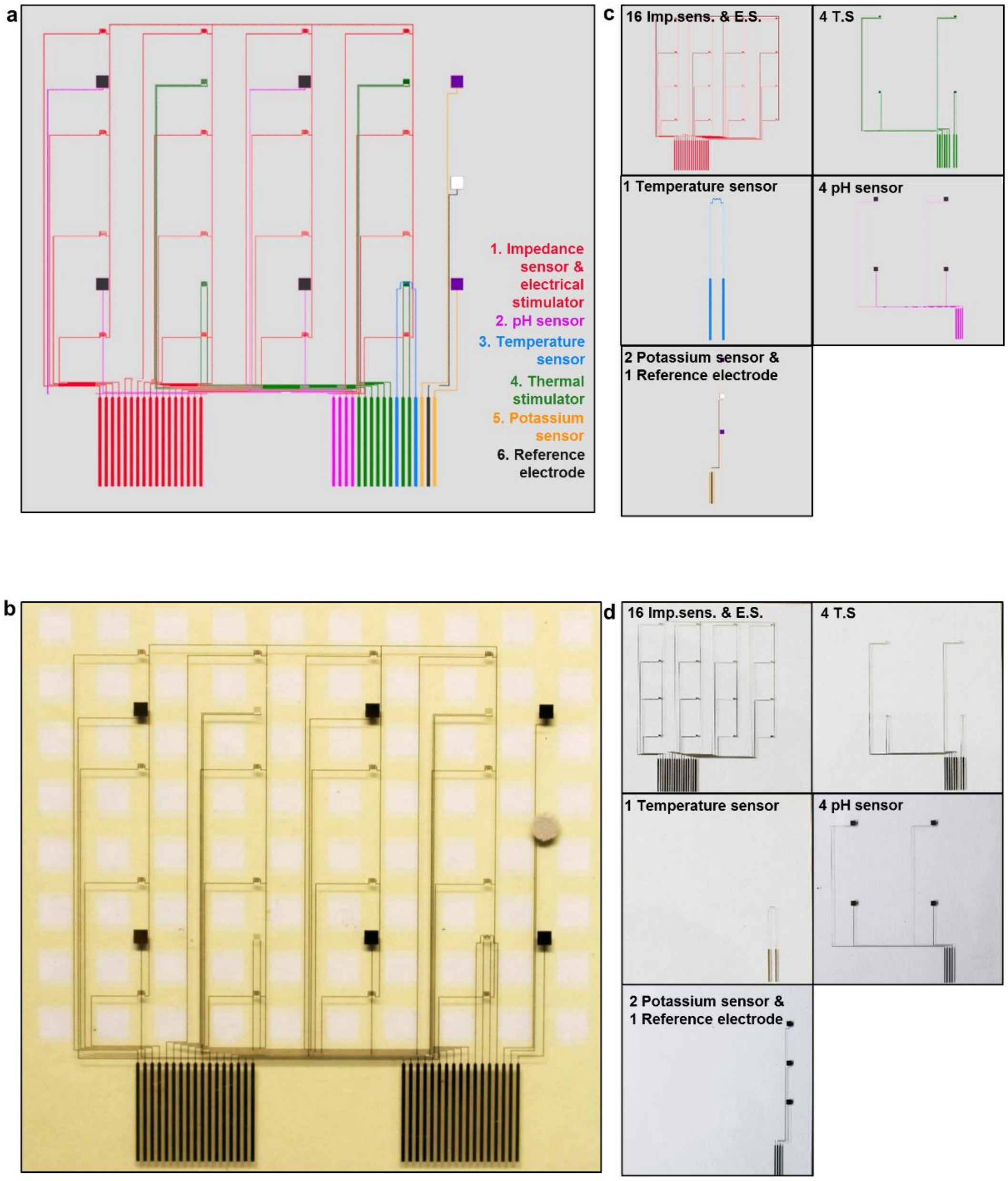

Supplementary Figure 3. Layout of the integrated array of sensors and stimulators in (a) AutoCAD designs and (b) photographic images. Each device (16 impedance sensors and electrical stimulators, 4 thermal stimulators, 1 temperature sensor, 4 pH sensors, 2 potassium working electrodes, and 1 reference electrode) is shown in (c) AutoCAD designs and (d) photographic images.

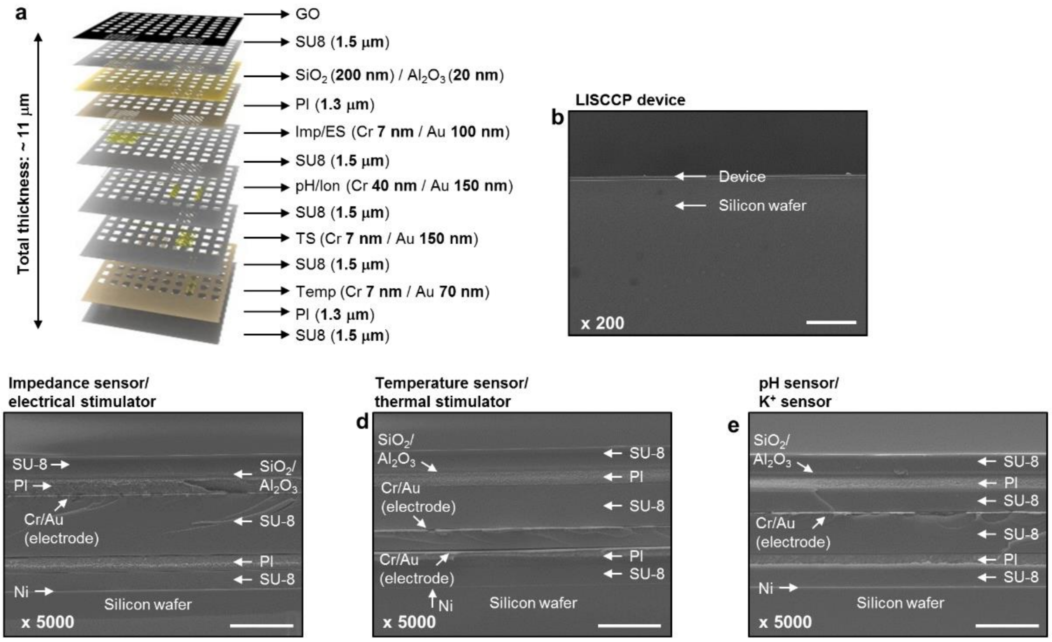

Supplementary Figure 4. Thickness information of the integrated ultrathin sensors and stimulators. **a**, exploded view of sensors and stimulators with thickness information for each layer. The total thickness of the sensors and stimulators including the encapsulation layers is ~11 mm. **b-e**, scanning electron microscopic images of the cross-sections for (b) the device, (c) impedance sensor/electrical stimulator, (d) temperature sensor and thermal stimulator, and (e) pH sensor/K^+ sensor. Scale bars for b: 100 μm and for c-e: 5 μm.

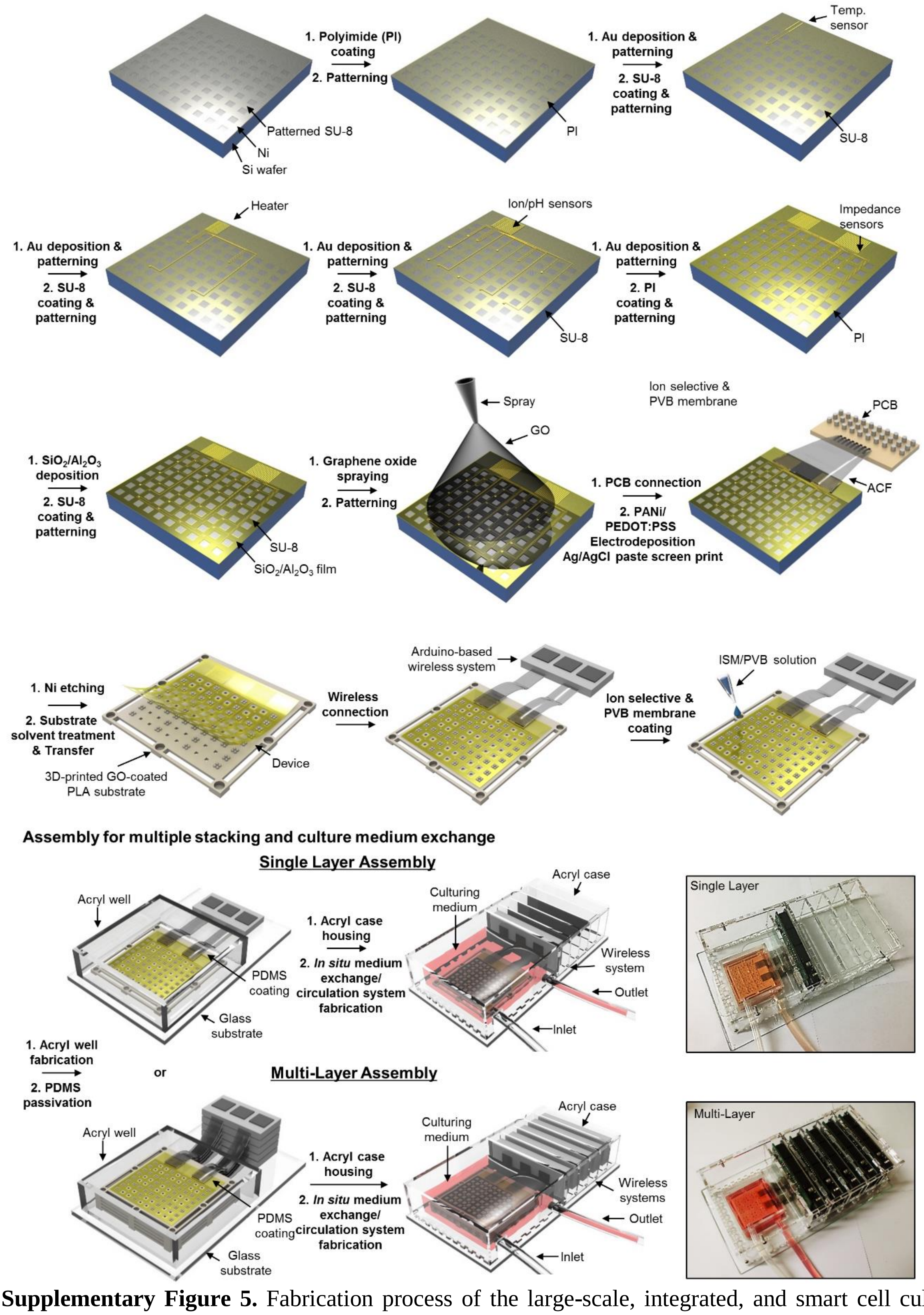

Supplementary Figure 5. Fabrication process of the large-scale, integrated, and smart cell culture platform.

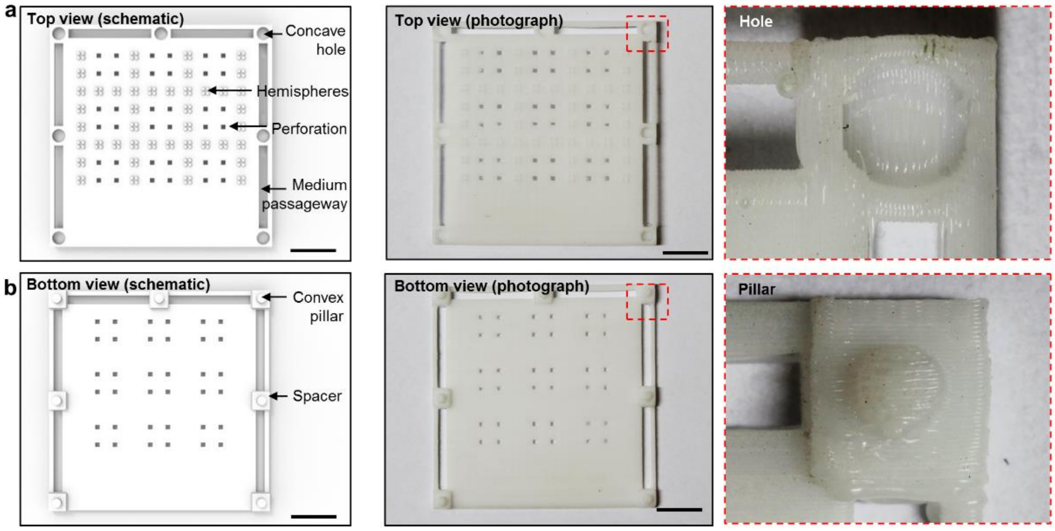

Supplementary Figure 6. Schematic illustration (left) and photographic images (center and right) of the 3D-printed PLA engineered substrate from (a) top and (b) bottom perspectives. Scale bar: 10 mm.

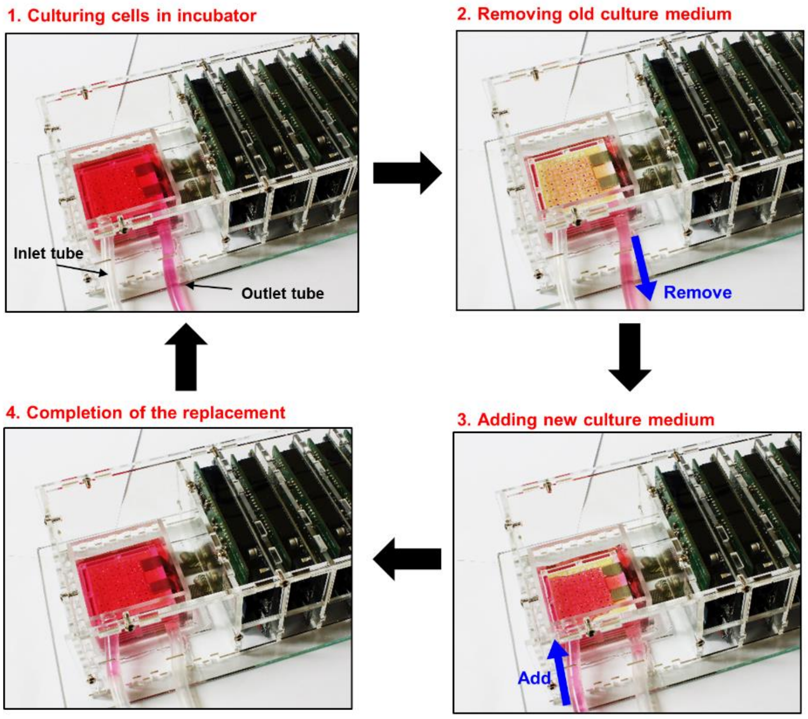

Supplementary Figure 7. Photographic images showing the process of the *in situ* culture medium exchange. The old culture medium is removed from the outlet tube, and fresh culture medium is added into an acryl well *via* the inlet tube.

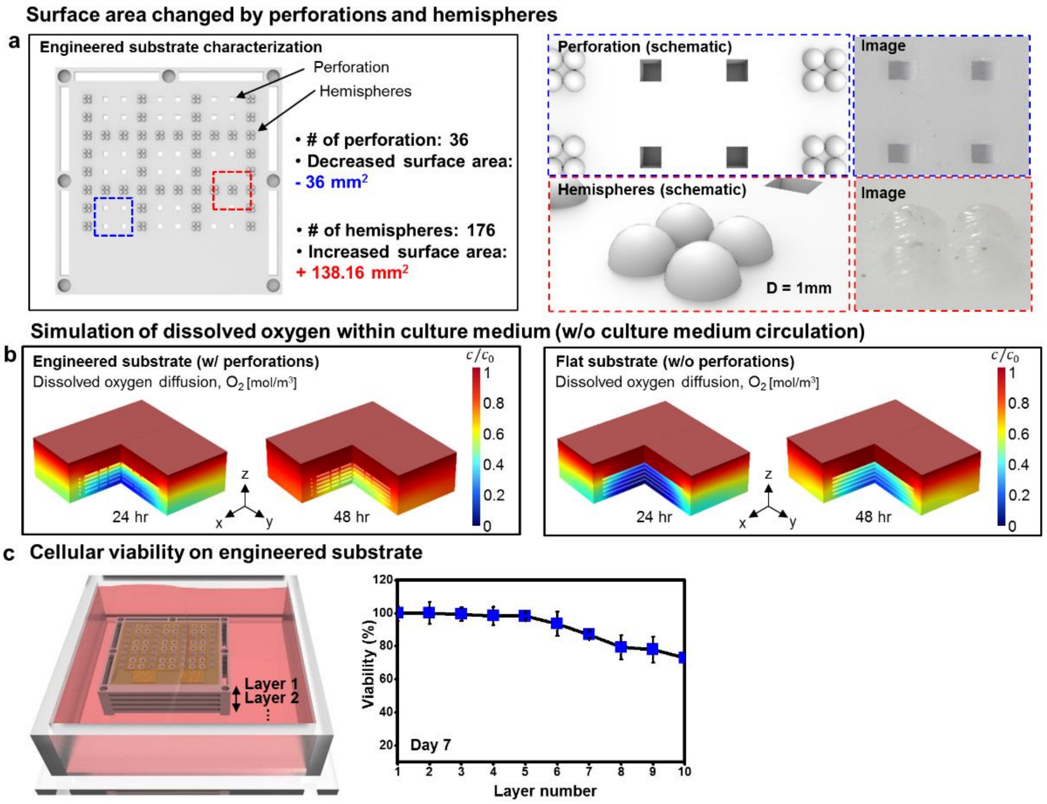

Supplementary Figure 8. Details of the PLA engineered substrate. **a**, Numerical information of the perforation and the protruded hemisphere designs along with their magnified photographic images. **b**, FEM analysis of dissolved oxygen distribution within culture medium with and without perforation in an engineered substrate. **c**, Viability of cells on the engineered substrates assembled into 10 layers (n=4, mean±s.d.).

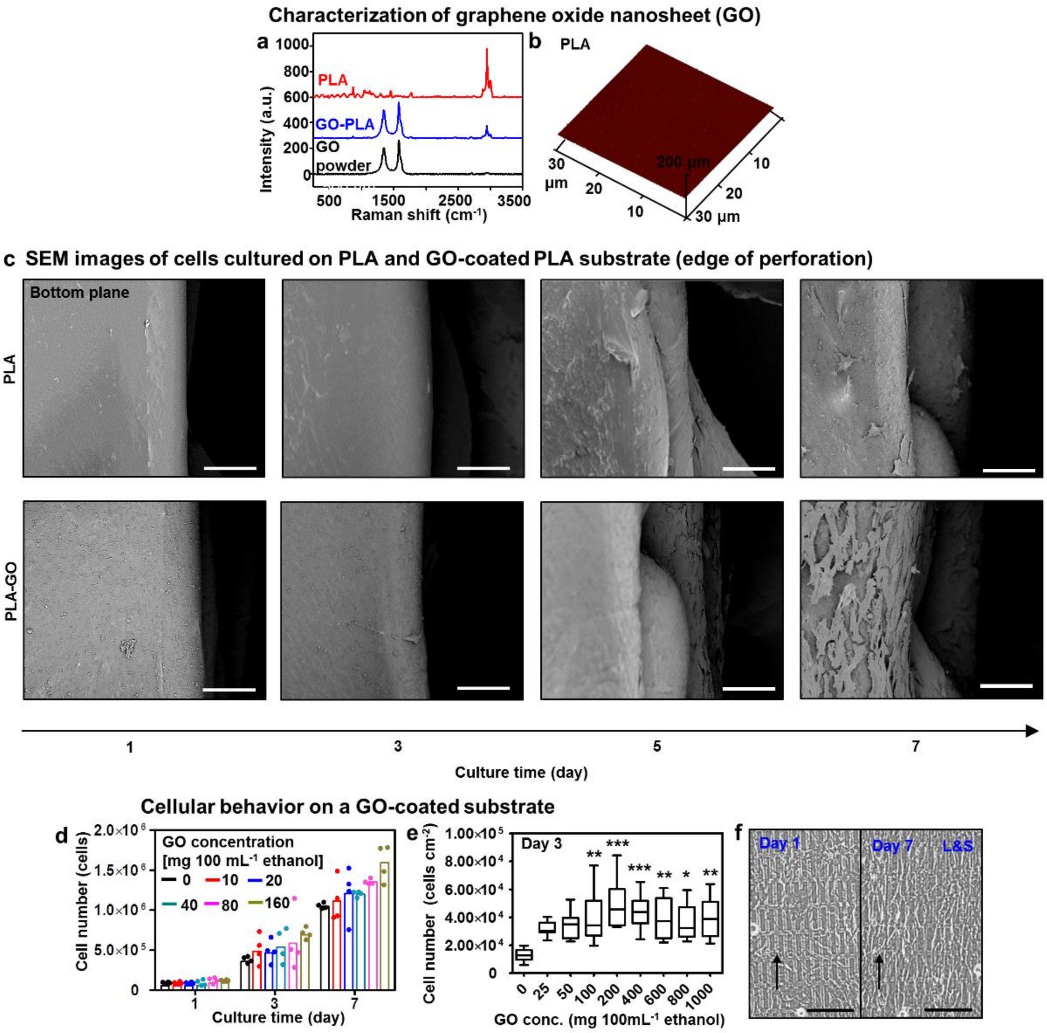

Supplementary Figure 9. Characterizations of graphene oxide nanosheet (GO)-coated 3D printed PLA substrate. a, Raman spectrum of the bare PLA substrate, a GO-coated PLA substrate, and GO powder. **b**, AFM image of the bare PLA substrate. **c**, SEM images of cells cultured on the edges of perforations in PLA and GO-coated PLA substrates. More C2C12 cells are adhered onto the GO-coated PLA substrate. Scale bar: 100 μm. **d**, Plot showing the number of cells compared to the concentration of GO coating. (n=4, mean) **e**, the number of C2C12 cells after 3-day culture on PLA coated with GO of a wide range of concentrations to examine the effect of GO concentration on the cell proliferation. (n=8, Box: median; 25th to 75th percentiles, Whiskers: min to max, *P<0.05, **P<0.01, ***P<0.001 versus zero concentration, ANOVA with Bonferroni's post-test) **f**, Optical microscopic image of cellular alignment of C2C12 cells induced by patterning GO into line and space. Scale bar: 50 μm.

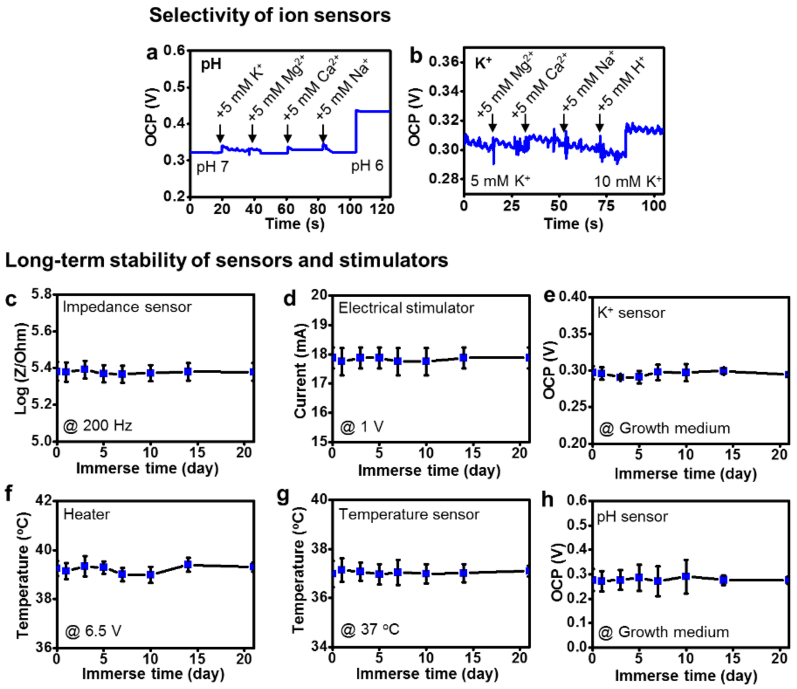

Supplementary Figure 10. Selectivity tests for (a) pH and (b) K^+ sensors. The interfering cations such as magnesium, calcium, sodium, potassium, and hydrogen ions are used. The long-term stability of the (c) impedance sensor, (d) electrical stimulator, (e) K^+ sensor, (f) heater, (g) temperature sensor, and (h) pH sensor in culture medium inside the CO_2 incubator for 3 weeks (n=4, mean±s.d.). For the long-term stability test, the devices were immersed in growth medium without culturing cells, and the medium was replaced every two days.

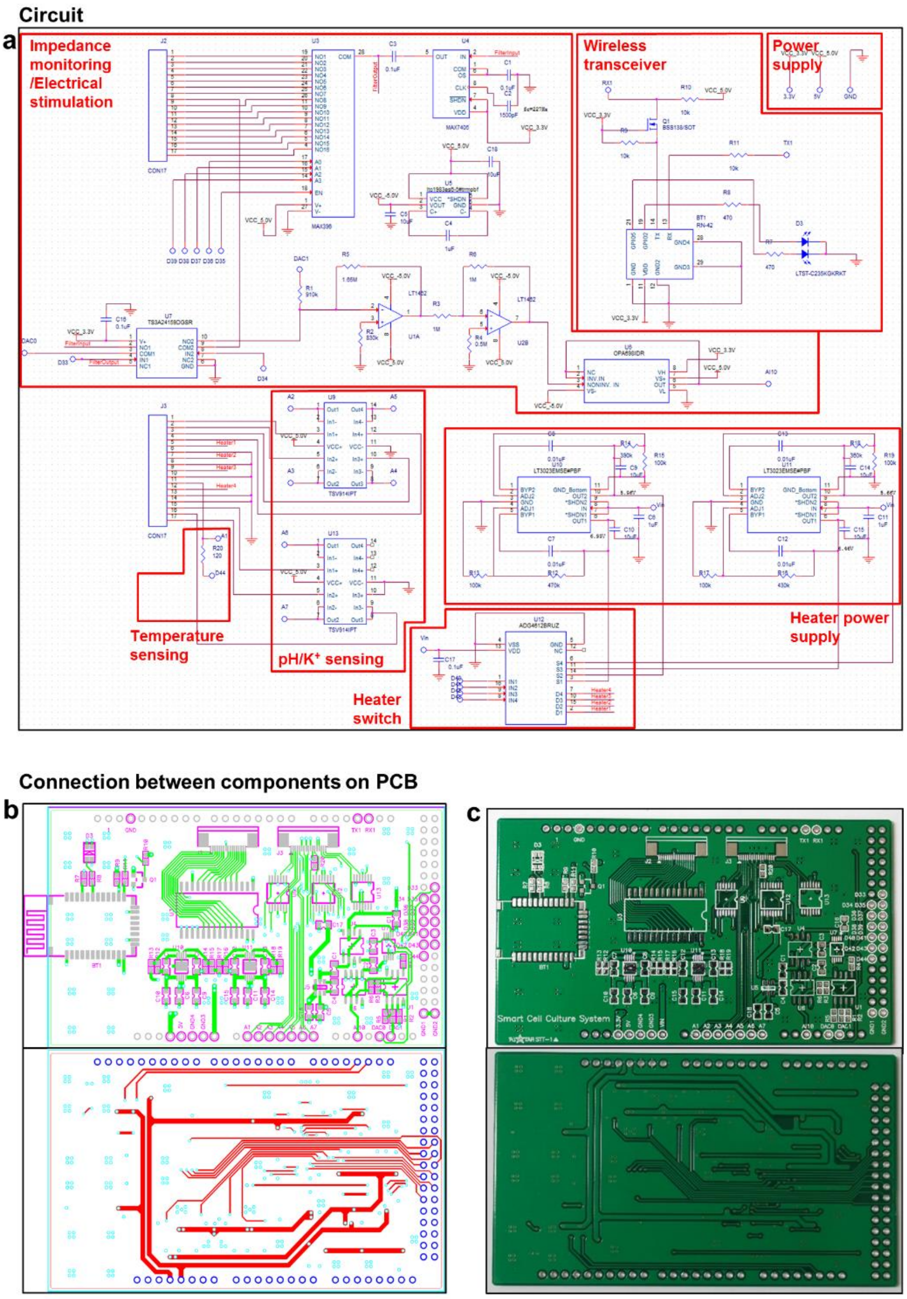

Supplementary Figure 11. Schematic illustrations of (a) circuits and (b) PCB designs with (c) photographic images.

Electronic components

Symbol	Role of the component	Model
U1A, U2B	Transimpedance amplifier and inverter for impedance sensing	LT1462
U3	Multiplexer for addressing each sensor/actuator for impedance sensing/electrical stimulation	MAX396
U4	Low-pass filter for smoothening the input sinusoidal signal during impedance sensing	MAX7405
U5	Regulator for supplying -5V to the transimpedance amplifier	LTC1983
U6	Voltage limiting operational amplifier (Op-amp) for protecting the ADC	OPA698
U7,13	Switching components for swapping between the modes of impedance sensing and electrical stimulation	TS3A24159
U9, U13	Op-amp array for constructing voltage follower circuit	TSV914
U10,11	Voltage regulator for providing designated voltage to the heaters of different resistance	LT3023
U12	Switching components for selecting the heaters to turn on	ADG4612
J2,3	Connector for making electrical interconnection between the PCB board and the cell culture platform	0527461771
Q1	Level shifter	BSS138
D3	LEDs for visualizing the status of data transmission	LTST-C235KGKRKT
BT1	Bluetooth module for wireless data transmission	RN-42
Rx	Resistor	-
Cx	Capacitor	-

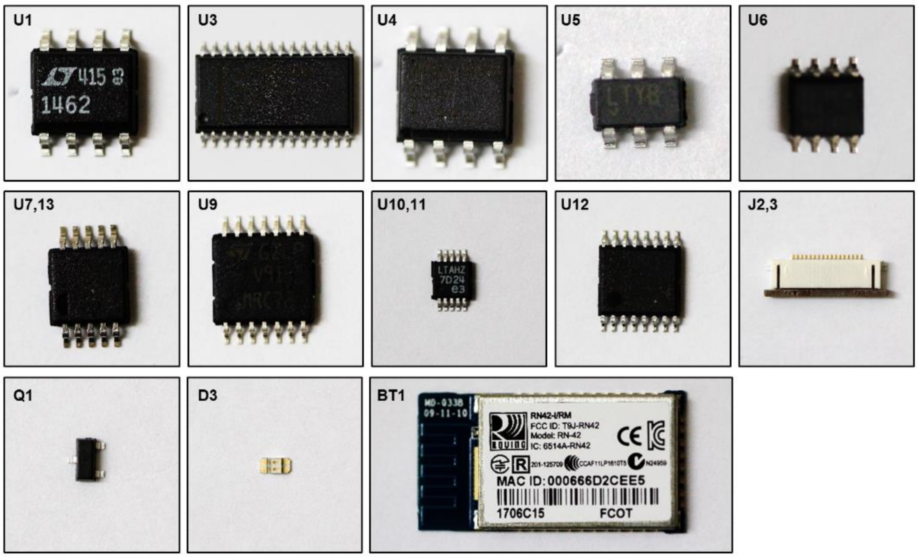

Supplementary Figure 12. Detailed descriptions of the electronic components used for the wireless system.

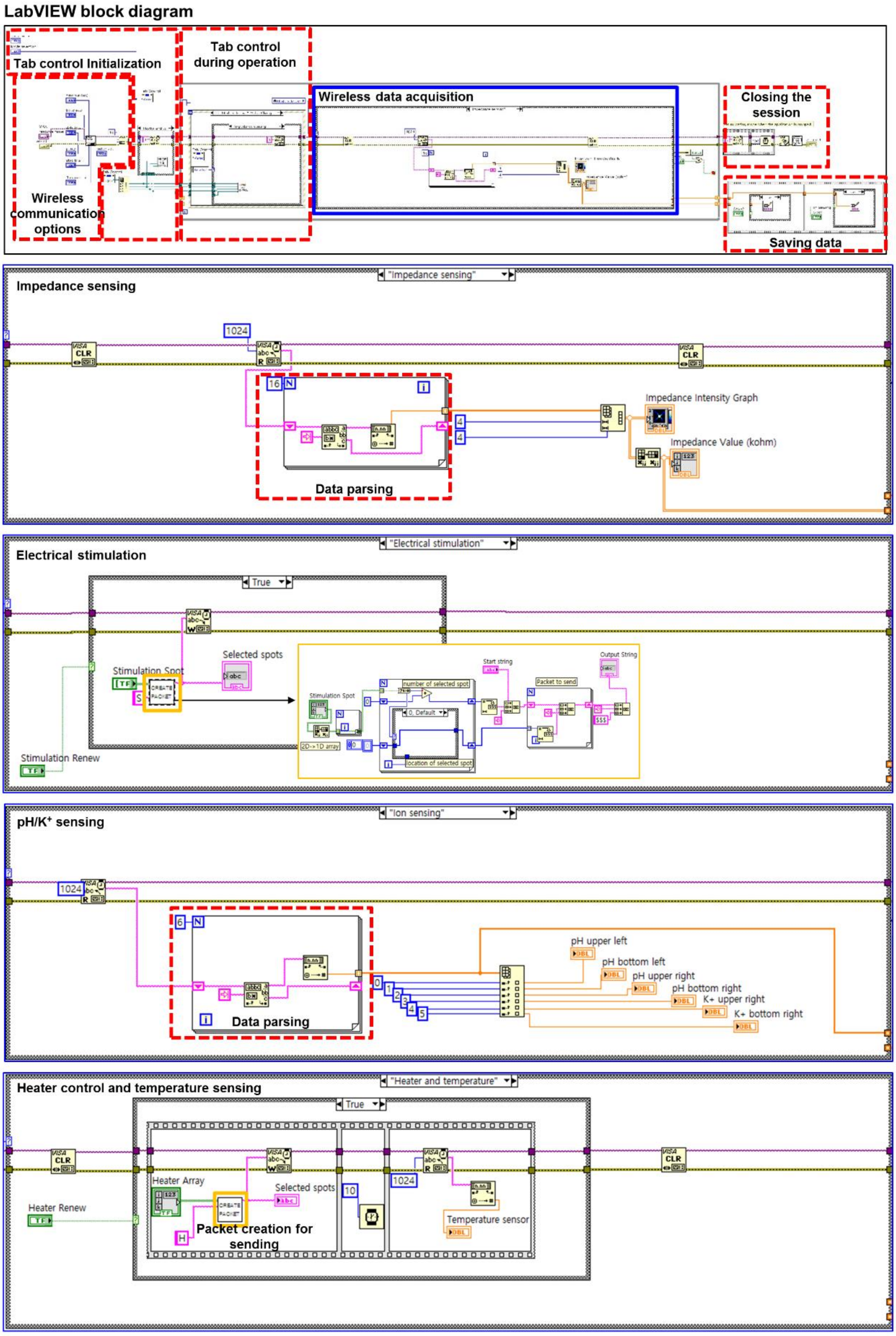

Supplementary Figure 13. LabVIEW block diagrams for the LISCCP control program.

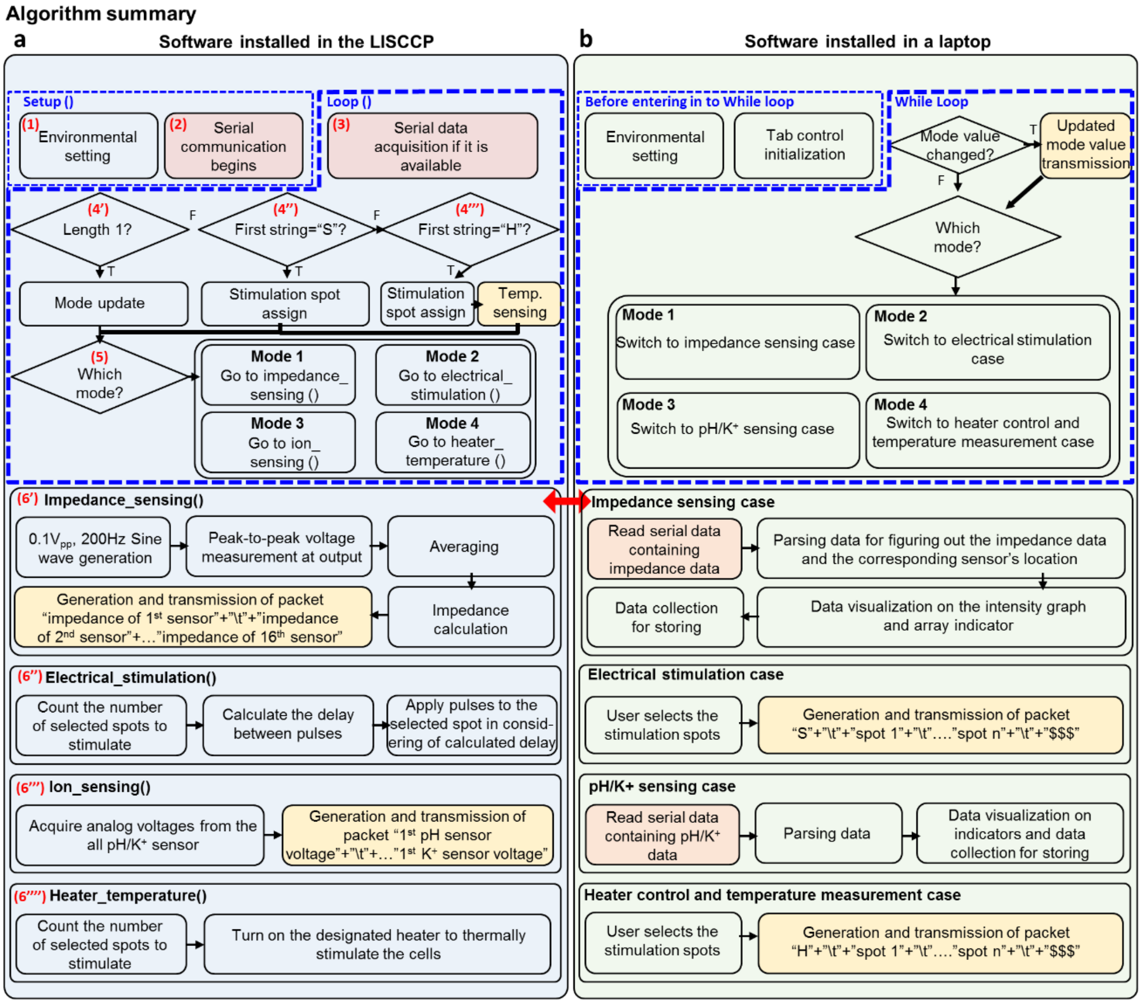

Supplementary Figure 14. Summary of the algorithm to measure and actuate the sensor and stimulator. **a**, software for the automated operation installed in LISCCP. LISCCP is initialized through the step (1) and (2). Then, the software initiated the loop and waits (3) for the command coming from the software installed in a laptop controlled by users. Depending on the case (4', 4'', 4'''), the software conducts the designated functions such as mode update, stimulation spot assign, and stimulation spot assign. Once this step (5) is finished, the software conducts either impedance sensing (6'), electrical stimulation (6''), ion sensing (6''') or temperature measurement (6'''') depending on the command received. **b**, a separate software installed in a laptop to control the LISCCP as well as to acquire data. Once users select a mode from the four possible modes, this information is conveyed to the LISCCP. Depending on the selected mode, the software's front panel and the functions being called are changed. In case of impedance sensing or pH/K^+ sensing mode, the designated sensors in LISCCP measure the designated data and the software wirelessly reads this data and visualizes them through the indicators. In case of electrical stimulation and heater control mode, actuators in the LISCCP are controlled by the commands sent by the software.

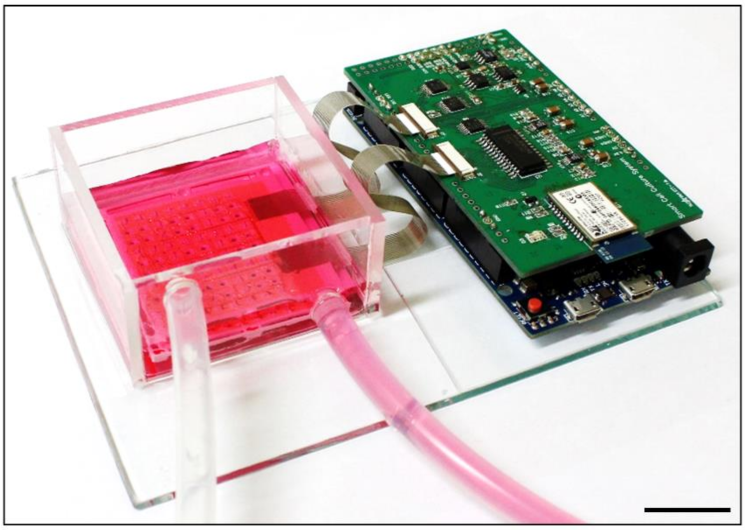

Supplementary Figure 15. Photographic image of the single-layered cell culture platform (CCP). Scale bar: 2 cm.

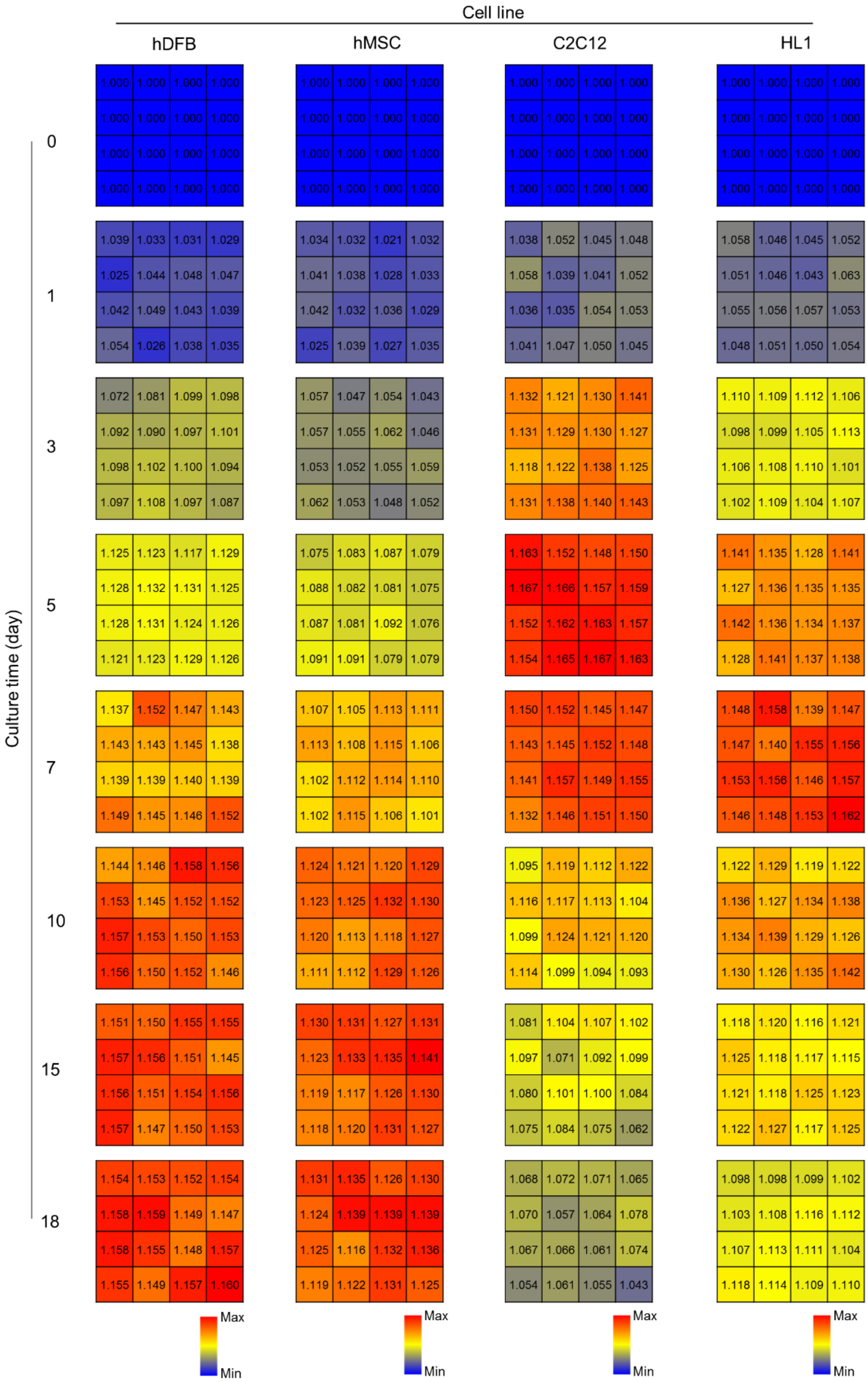

Supplementary Figure 16. Array impedance mappings of hDFB, hMSC, C2C12, and HL-1 cultured without any electrical or thermal stimulations on single-layered CCPs.

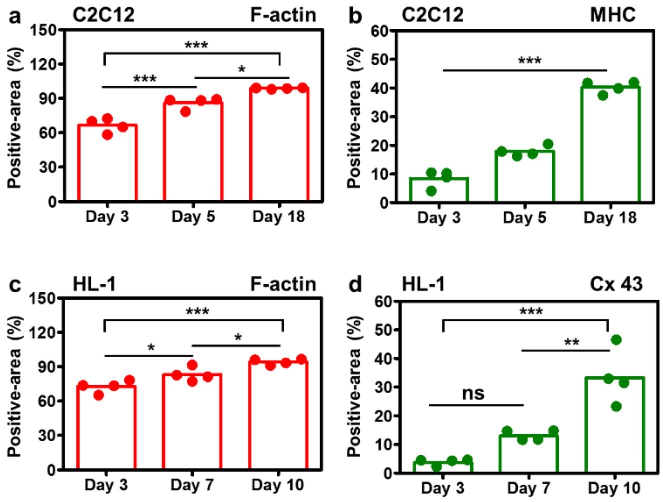

Supplementary Figure 17. Quantitative descriptions on immunostaining images (Figure 4i,k) of C2C12 and HL-1 differentiations. a-b, (a) F-actin- and (b) myosin heavy chain-positive areas in the C2C12 culture. **c-d**, (c) F-actin- and (d) connexin 43-positive area of the HL-1 culture. (n=4, mean, *P<0.05, **P<0.01, ***P<0.001, ns, not significant, ANOVA with Bonferroni's post-test)

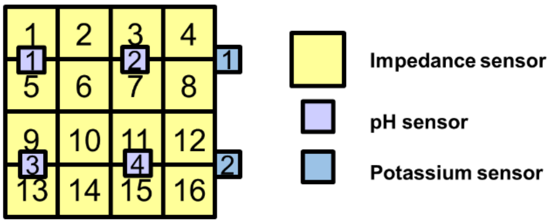

Layer 1

Day	Imp. 1	Imp. 2	Imp. 3	Imp. 4	Imp. 5	Imp. 6	Imp. 7	Imp. 8	Imp. 9	Imp. 10	Imp. 11	Imp. 12	Imp. 13	Imp. 14	Imp. 15	Imp. 16	pH 1	pH 2	pH 3	pH 4	K+ 1	K+ 2
0	1.000	1.000	1.000	1.000	1.000	1.000	1.000	1.000	1.000	1.000	1.000	1.000	1.000	1.000	1.000	1.000	7.33	7.30	7.35	7.33	5.33	5.34
1	1.106	1.105	1.109	1.095	1.109	1.110	1.104	1.109	1.095	1.095	1.105	1.116	1.108	1.114	1.100	1.114	7.24	7.20	7.25	7.23	5.30	5.31
3	1.143	1.142	1.145	1.131	1.146	1.147	1.141	1.146	1.132	1.132	1.142	1.153	1.145	1.151	1.137	1.151	7.12	7.08	7.14	7.11	5.26	5.27
5	1.180	1.178	1.182	1.168	1.183	1.184	1.178	1.183	1.169	1.169	1.179	1.190	1.182	1.188	1.174	1.188	7.06	7.02	7.08	7.05	5.16	5.17
7	1.230	1.213	1.206	1.215	1.223	1.252	1.228	1.226	1.212	1.217	1.218	1.217	1.205	1.222	1.217	1.213	7.00	6.96	7.02	6.99	5.00	5.01
10	1.166	1.164	1.168	1.154	1.169	1.169	1.164	1.169	1.155	1.154	1.164	1.176	1.167	1.173	1.159	1.174	6.97	6.93	6.99	6.97	4.83	4.84
14	1.135	1.134	1.137	1.123	1.138	1.139	1.133	1.138	1.124	1.124	1.134	1.145	1.137	1.143	1.129	1.143	6.95	6.91	6.96	6.94	4.80	4.81
18	1.113	1.112	1.115	1.101	1.116	1.117	1.111	1.116	1.102	1.102	1.112	1.123	1.115	1.121	1.107	1.121	6.95	6.91	6.96	6.94	4.87	4.88

Layer 2

Day	Imp. 1	Imp. 2	Imp. 3	Imp. 4	Imp. 5	Imp. 6	Imp. 7	Imp. 8	Imp. 9	Imp. 10	Imp. 11	Imp. 12	Imp. 13	Imp. 14	Imp. 15	Imp. 16	pH 1	pH 2	pH 3	pH 4	K+ 1	K+ 2
0	1.000	1.000	1.000	1.000	1.000	1.000	1.000	1.000	1.000	1.000	1.000	1.000	1.000	1.000	1.000	1.000	7.33	7.36	7.35	7.32	5.32	5.32
1	1.116	1.099	1.107	1.087	1.111	1.108	1.097	1.107	1.108	1.099	1.107	1.100	1.111	1.112	1.090	1.087	7.32	7.26	7.25	7.23	5.29	5.29
3	1.153	1.136	1.144	1.123	1.148	1.145	1.134	1.144	1.145	1.136	1.144	1.157	1.148	1.149	1.127	1.124	7.17	7.15	7.13	7.11	5.25	5.25
5	1.190	1.173	1.181	1.160	1.185	1.182	1.171	1.181	1.182	1.173	1.181	1.194	1.185	1.186	1.164	1.161	7.10	7.07	7.08	7.04	5.12	5.15
7	1.229	1.227	1.215	1.213	1.221	1.239	1.218	1.218	1.203	1.206	1.225	1.214	1.217	1.191	1.220	1.203	7.06	7.02	7.02	6.98	4.96	4.99
10	1.175	1.158	1.166	1.146	1.171	1.168	1.157	1.167	1.167	1.158	1.166	1.160	1.170	1.172	1.149	1.147	7.03	6.99	6.99	6.95	4.78	4.82
14	1.145	1.128	1.136	1.115	1.140	1.137	1.126	1.136	1.137	1.128	1.136	1.149	1.162	1.164	1.141	1.139	6.99	6.96	6.96	6.93	4.76	4.79
18	1.123	1.106	1.114	1.093	1.118	1.115	1.104	1.114	1.115	1.106	1.114	1.127	1.110	1.112	1.119	1.117	6.98	6.96	6.96	6.93	4.86	4.86

Layer 3

Day	Imp. 1	Imp. 2	Imp. 3	Imp. 4	Imp. 5	Imp. 6	Imp. 7	Imp. 8	Imp. 9	Imp. 10	Imp. 11	Imp. 12	Imp. 13	Imp. 14	Imp. 15	Imp. 16	pH 1	pH 2	pH 3	pH 4	K+ 1	K+ 2
0	1.000	1.000	1.000	1.000	1.000	1.000	1.000	1.000	1.000	1.000	1.000	1.000	1.000	1.000	1.000	1.000	7.31	7.27	7.32	7.36	5.33	5.31
1	1.109	1.093	1.093	1.108	1.103	1.093	1.108	1.102	1.100	1.101	1.105	1.105	1.102	1.107	1.095	1.094	7.22	7.17	7.22	7.26	5.31	5.29
3	1.146	1.130	1.130	1.145	1.140	1.130	1.145	1.139	1.137	1.137	1.142	1.142	1.139	1.144	1.132	1.131	7.10	7.03	7.09	7.11	5.26	5.24
5	1.182	1.167	1.167	1.182	1.177	1.167	1.182	1.176	1.174	1.174	1.178	1.179	1.176	1.181	1.169	1.168	7.05	6.96	7.03	7.04	5.10	5.09
7	1.219	1.213	1.214	1.213	1.217	1.215	1.214	1.211	1.213	1.215	1.218	1.215	1.216	1.216	1.212	1.219	6.99	6.91	6.98	7.00	4.93	4.96
10	1.168	1.152	1.152	1.168	1.163	1.153	1.168	1.162	1.160	1.160	1.164	1.164	1.162	1.166	1.154	1.154	6.96	6.88	6.95	6.97	4.76	4.78
14	1.138	1.122	1.122	1.137	1.132	1.122	1.137	1.131	1.129	1.129	1.134	1.134	1.131	1.136	1.124	1.123	6.93	6.85	6.91	6.93	4.74	4.76
18	1.116	1.100	1.100	1.115	1.110	1.100	1.115	1.109	1.107	1.107	1.112	1.112	1.109	1.114	1.102	1.101	6.93	6.85	6.90	6.92	4.83	4.84

Layer 4

Day	Imp. 1	Imp. 2	Imp. 3	Imp. 4	Imp. 5	Imp. 6	Imp. 7	Imp. 8	Imp. 9	Imp. 10	Imp. 11	Imp. 12	Imp. 13	Imp. 14	Imp. 15	Imp. 16	pH 1	pH 2	pH 3	pH 4	K+ 1	K+ 2
0	1.000	1.000	1.000	1.000	1.000	1.000	1.000	1.000	1.000	1.000	1.000	1.000	1.000	1.000	1.000	1.000	7.33	7.31	7.29	7.29	5.33	5.33
1	1.107	1.100	1.104	1.105	1.086	1.101	1.099	1.087	1.106	1.099	1.103	1.101	1.092	1.086	1.108	1.086	7.23	7.22	7.19	7.20	5.31	5.30
3	1.144	1.137	1.141	1.142	1.123	1.138	1.136	1.154	1.143	1.136	1.140	1.138	1.129	1.123	1.145	1.123	6.97	7.10	7.07	7.08	5.26	5.26
5	1.181	1.174	1.178	1.179	1.160	1.175	1.173	1.191	1.180	1.173	1.177	1.175	1.166	1.160	1.182	1.160	6.90	7.03	7.02	7.01	5.16	5.13
7	1.224	1.223	1.230	1.189	1.231	1.240	1.219	1.202	1.219	1.217	1.214	1.232	1.227	1.224	1.217	1.237	6.86	6.97	6.95	6.95	5.01	4.97
10	1.167	1.160	1.163	1.165	1.145	1.160	1.159	1.177	1.165	1.159	1.163	1.160	1.152	1.146	1.168	1.145	6.87	6.94	6.92	6.92	4.83	4.79
14	1.136	1.129	1.133	1.134	1.115	1.130	1.128	1.146	1.135	1.128	1.132	1.130	1.121	1.115	1.137	1.115	6.86	6.92	6.90	6.90	4.81	4.77
18	1.114	1.107	1.111	1.112	1.093	1.108	1.106	1.114	1.113	1.106	1.110	1.108	1.099	1.093	1.115	1.093	6.84	6.91	6.90	6.90	4.87	4.87

Layer 5

Day	Imp. 1	Imp. 2	Imp. 3	Imp. 4	Imp. 5	Imp. 6	Imp. 7	Imp. 8	Imp. 9	Imp. 10	Imp. 11	Imp. 12	Imp. 13	Imp. 14	Imp. 15	Imp. 16	pH 1	pH 2	pH 3	pH 4	K+ 1	K+ 2
0	1.000	1.000	1.000	1.000	1.000	1.000	1.000	1.000	1.000	1.000	1.000	1.000	1.000	1.000	1.000	1.000	7.28	7.31	7.30	7.36	5.33	5.31
1	1.086	1.104	1.100	1.086	1.102	1.106	1.106	1.103	1.103	1.100	1.107	1.098	1.089	1.098	1.096	1.093	7.19	7.21	7.20	7.26	5.31	5.28
3	1.123	1.141	1.137	1.123	1.139	1.143	1.143	1.140	1.140	1.137	1.144	1.134	1.126	1.135	1.133	1.130	7.07	6.96	7.08	7.00	5.26	5.24
5	1.160	1.178	1.174	1.160	1.176	1.180	1.180	1.177	1.177	1.174	1.181	1.171	1.163	1.172	1.170	1.167	7.01	6.89	7.02	6.93	5.11	5.07
7	1.234	1.232	1.214	1.211	1.216	1.220	1.209	1.207	1.230	1.219	1.212	1.212	1.213	1.204	1.211	1.211	6.95	6.85	6.96	6.89	4.98	4.91
10	1.146	1.164	1.160	1.146	1.162	1.166	1.165	1.162	1.162	1.159	1.167	1.157	1.149	1.157	1.156	1.153	6.92	6.83	6.94	6.87	4.80	4.74
14	1.115	1.133	1.129	1.115	1.131	1.135	1.135	1.132	1.132	1.129	1.136	1.126	1.118	1.127	1.125	1.122	6.90	6.84	6.91	6.83	4.78	4.72
18	1.093	1.111	1.107	1.093	1.109	1.113	1.113	1.110	1.110	1.107	1.114	1.104	1.096	1.105	1.103	1.100	6.90	6.82	6.91	6.82	4.86	4.81

1.00 Norm. imp. 1.25 — 7.4 pH 6.8 — 4.7 [K^+] mM 5.4

Supplementary Figure 18. Massive monitoring data of C2C12 cultured on the LISCCP.

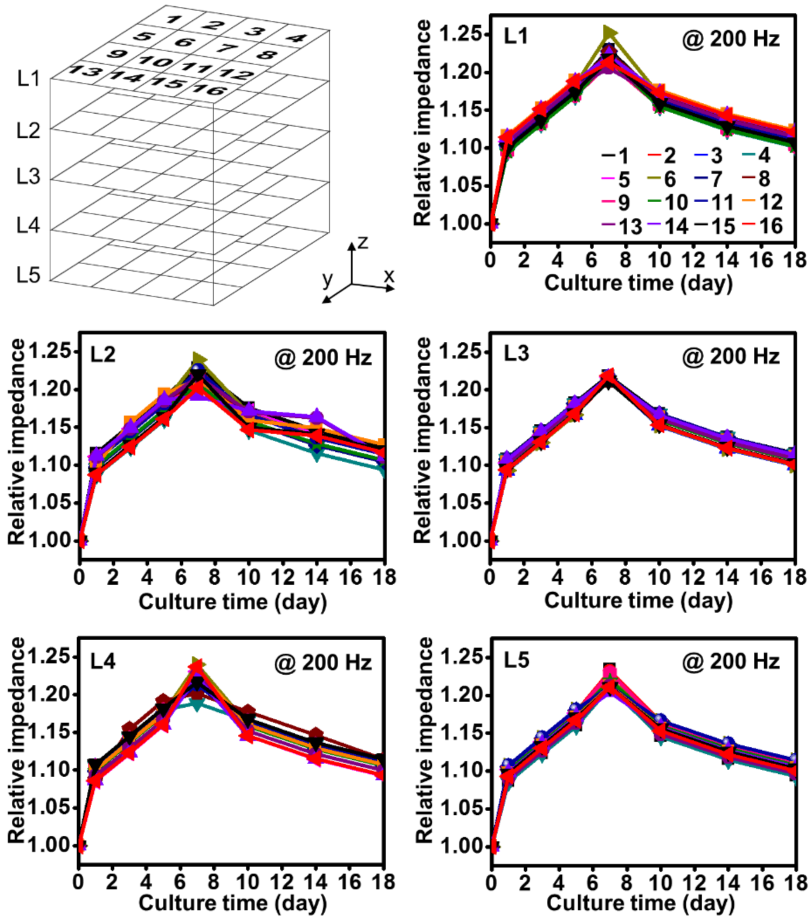

Supplementary Figure 19. Plots showing 3D multilayer impedance mapping of C2C12 without any stimulations.

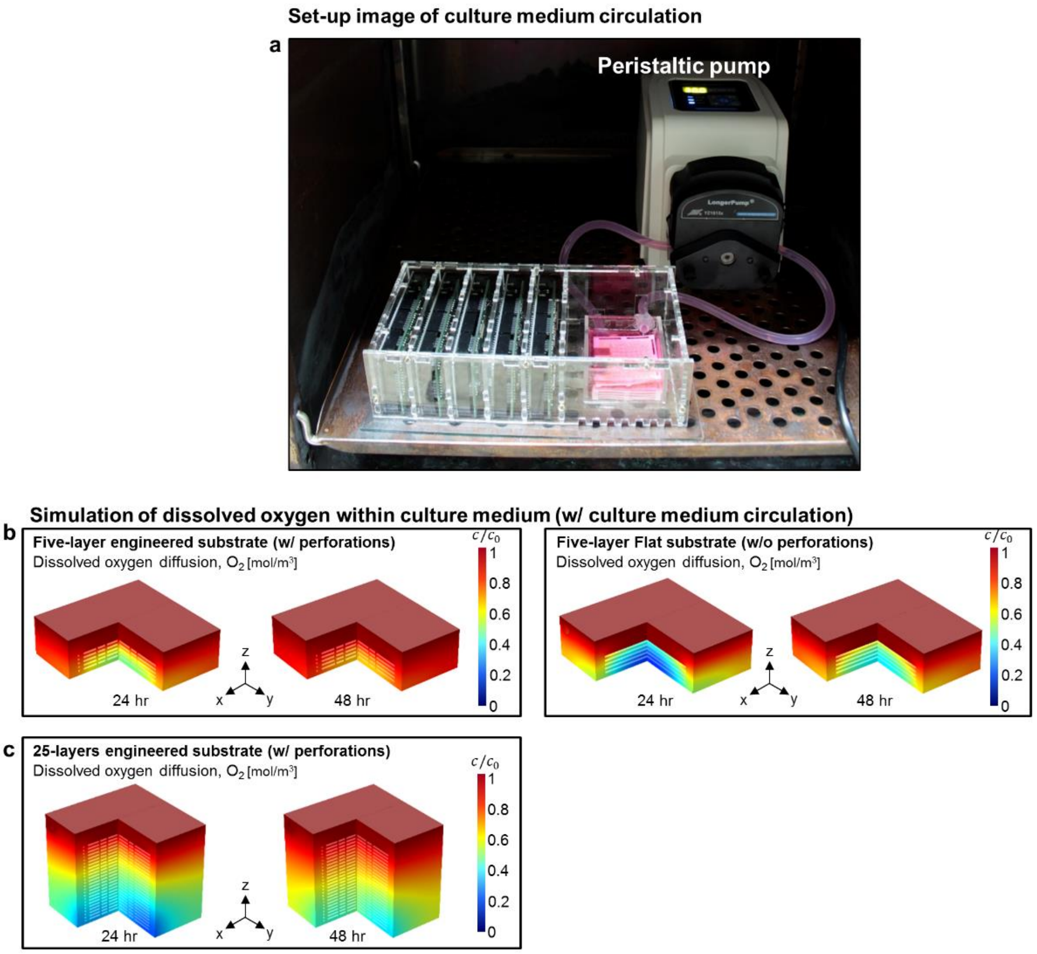

Supplementary Figure 20. Details of the culture medium circulation. **a**, Image of the culture medium circulation set-up in the CO_2 incubator **b**, FEM analysis of the dissolved oxygen distribution with continuous culture medium circulation on (left) the five-layer engineered substrate and (right) five-layer flat substrate. **c**, FEM analysis of the dissolved oxygen distribution with continuous culture medium circulation on 25-layer engineered substrate.

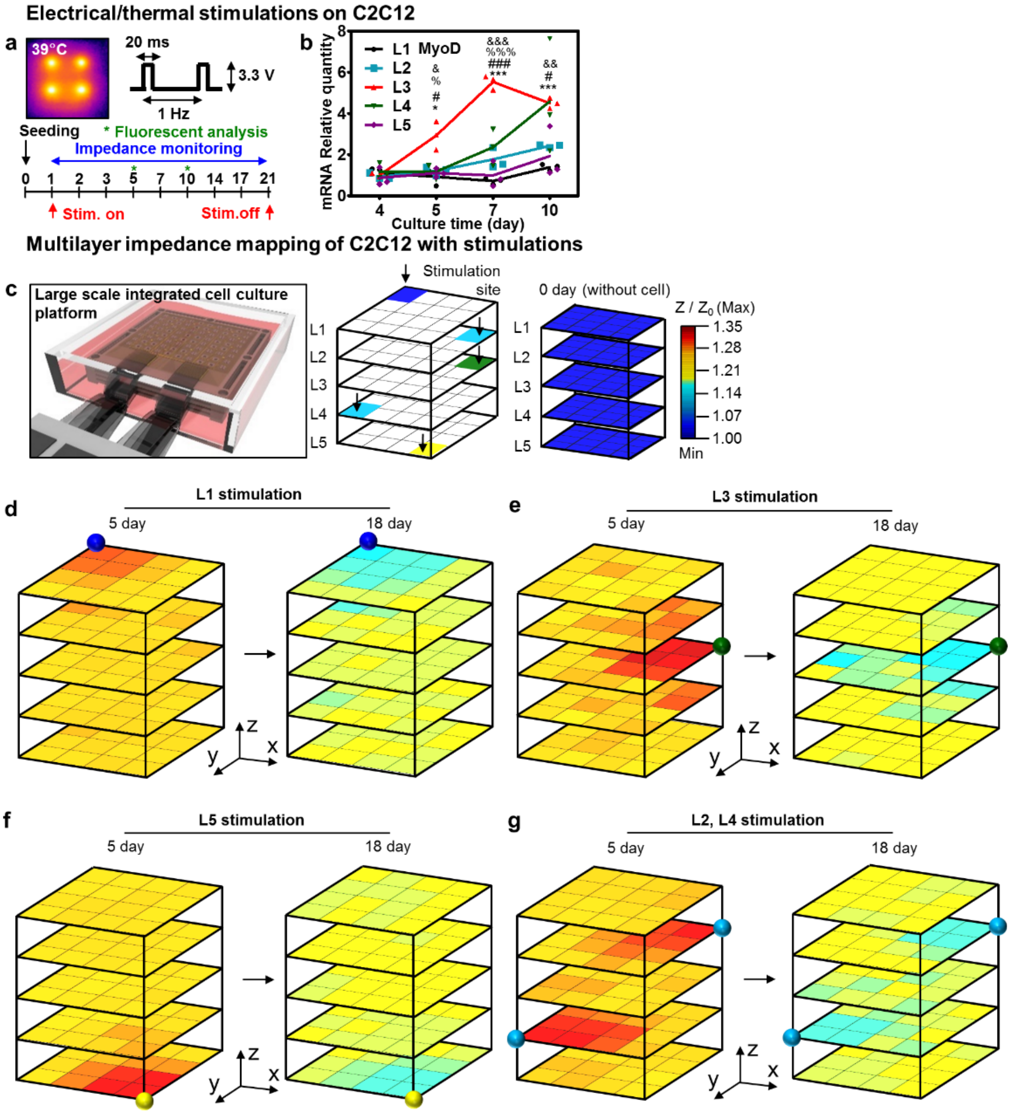

Supplementary Figure 21. 3D multilayer impedance mapping of C2C12 with localized electrical and thermal stimulations. a, Time table (bottom) for electrical stimulation (ES) and thermal stimulation (TS) of C2C12 myoblasts with an infrared image of TS (top left) and key parameters of ES (top right). Fluorescent staining analysis (green asterisk) was performed for F-actin on day 5 and for myotubes on day 10. **b**, The mRNA analysis of MyoD gene for electrical and thermal stimulations onto C2C12 cultured at 5-layer substrates. (n=3, mean, L3 compared to L1, *P<0.05, ***P<0.001; L3 compared to L2, #<0.05, ##P<0.001; L3 compared to L4, %P<0.05, %%%P<0.001; L3 compared to L5, &P<0.05, &&P<0.01, &&&P<0.001, ANOVA with Bonferroni's post-test). **c**, Schematic illustration of the cell culture and the locations of stimulations. **d-g**, 3D multilayer color mappings of impedance measurements on (d) L1 stimulation, (e) L3 stimulation, (f) L5 stimulation, and (g) both L2 and L4 stimulations. The dot indicates the stimulation site.

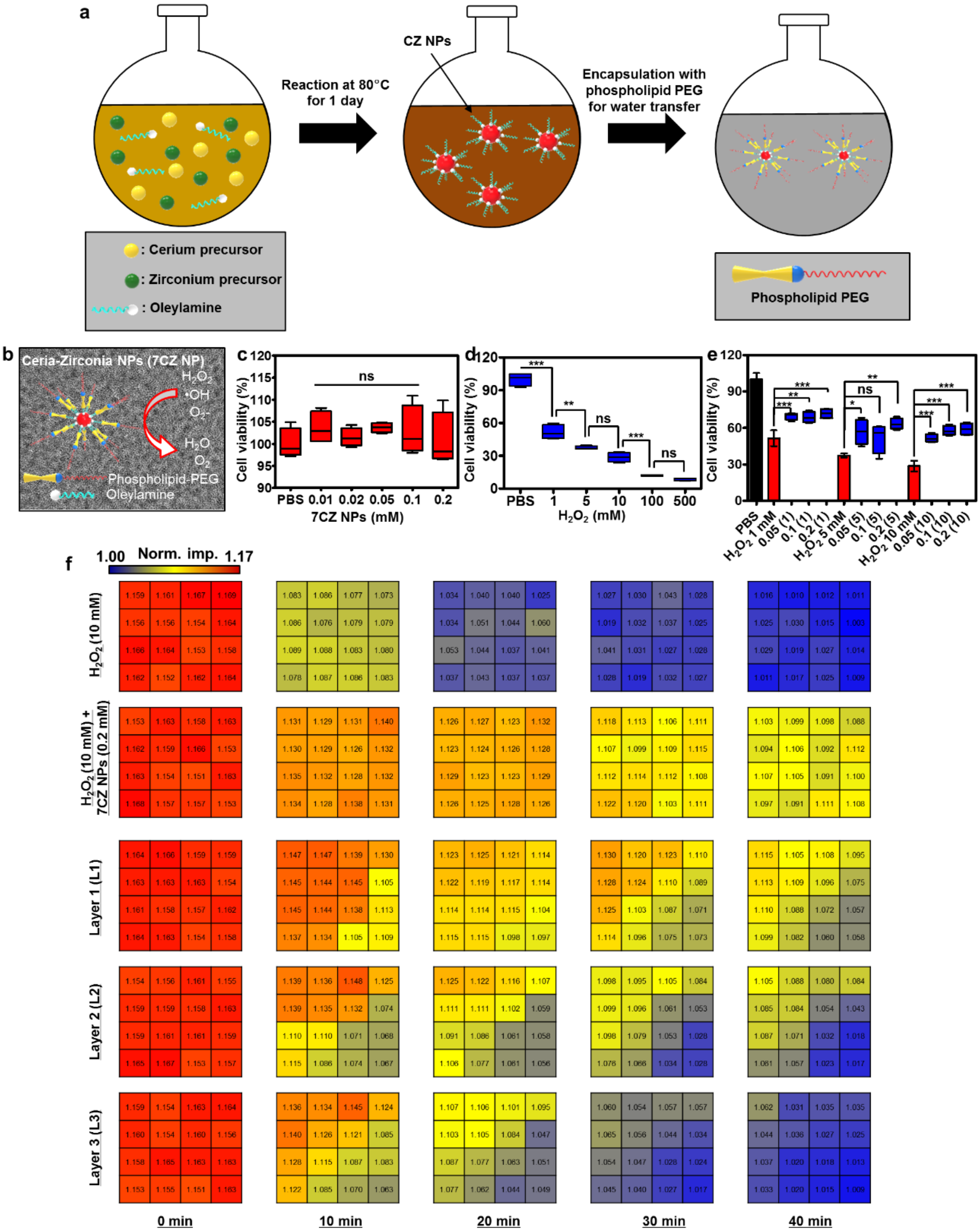

Supplementary Figure 22. Characterizations of ceria-zirconia nanoparticles (7CZ NP). a, Schematic illustration of 7CZ NP synthesis. **b**, TEM image of 7CZ (background) and a schematic illustration of 7CZ scavenging reactive oxygen species. **c-e**, Cellular viabilities of HL-1 cells treated with (c) H_2O_2 (n=4, Box: median; 25th to 75th percentiles, Whiskers: min to max, ns, not significant, ANOVA), (d) 7CZ NPs, and (e) a combination of H_2O_2 and 7CZ NPs. (n=4, ***P<0.001, **P<0.01, *P<0.05, ANOVA) **f**, Detailed impedance color maps for HL-1 treated with H_2O_2 and 7CZ NPs for two single-layered CCPs and one 3-layered CCP.

a 1.00 Norm. imp. 1.19

PBS

Time (min)	Imp. 1	Imp. 2	Imp. 3	Imp. 4	Imp. 5	Imp. 6	Imp. 7	Imp. 8	Imp. 9	Imp. 10	Imp. 11	Imp. 12	Imp. 13	Imp. 14	Imp. 15	Imp. 16
0	1.154	1.150	1.142	1.149	1.157	1.161	1.146	1.142	1.157	1.156	1.151	1.154	1.150	1.149	1.151	1.146
5	1.140	1.158	1.143	1.140	1.151	1.152	1.151	1.146	1.152	1.146	1.157	1.139	1.154	1.150	1.144	1.148
10	1.149	1.141	1.147	1.147	1.144	1.140	1.141	1.145	1.143	1.152	1.148	1.142	1.155	1.150	1.153	1.144
15	1.141	1.136	1.144	1.157	1.143	1.144	1.142	1.149	1.138	1.134	1.148	1.145	1.145	1.142	1.144	1.149
20	1.140	1.147	1.132	1.144	1.138	1.139	1.141	1.145	1.142	1.144	1.141	1.144	1.149	1.144	1.136	1.135
25	1.155	1.149	1.140	1.107	1.185	1.132	1.177	1.136	1.168	1.139	1.159	1.155	1.155	1.133	1.127	1.145
30	1.134	1.137	1.142	1.155	1.158	1.140	1.132	1.110	1.138	1.132	1.143	1.162	1.142	1.112	1.183	1.145
35	1.122	1.178	1.144	1.129	1.186	1.117	1.147	1.149	1.152	1.147	1.157	1.128	1.127	1.150	1.138	1.132
40	1.141	1.166	1.161	1.145	1.154	1.138	1.138	1.129	1.140	1.146	1.130	1.158	1.151	1.145	1.143	1.150

H2O2

Time (min)	Imp. 1	Imp. 2	Imp. 3	Imp. 4	Imp. 5	Imp. 6	Imp. 7	Imp. 8	Imp. 9	Imp. 10	Imp. 11	Imp. 12	Imp. 13	Imp. 14	Imp. 15	Imp. 16
0	1.155	1.150	1.156	1.152	1.155	1.152	1.147	1.151	1.149	1.144	1.136	1.145	1.147	1.152	1.150	1.153
5	1.128	1.128	1.128	1.128	1.125	1.124	1.128	1.128	1.129	1.127	1.128	1.128	1.128	1.127	1.130	1.128
10	1.098	1.110	1.103	1.098	1.101	1.103	1.106	1.117	1.100	1.100	1.104	1.103	1.099	1.107	1.106	1.110
15	1.080	1.081	1.078	1.073	1.075	1.079	1.080	1.074	1.084	1.077	1.081	1.077	1.077	1.080	1.079	1.074
20	1.057	1.064	1.065	1.053	1.068	1.058	1.071	1.072	1.041	1.059	1.046	1.059	1.058	1.062	1.061	1.075
25	1.038	1.031	1.032	1.038	1.043	1.022	1.026	1.034	1.051	1.042	1.043	1.039	1.040	1.042	1.034	1.029
30	1.034	1.012	1.026	1.014	1.024	1.018	1.019	1.012	1.020	1.042	1.035	1.010	1.014	1.042	1.015	0.999
35	0.992	0.992	1.010	1.009	0.994	1.009	1.013	0.987	1.013	1.038	1.009	1.039	1.013	1.009	0.994	0.992
40	1.007	1.010	1.003	1.009	1.012	1.011	1.012	1.008	1.009	1.006	1.005	1.006	1.012	1.007	1.007	1.011

ALS

Time (min)	Imp. 1	Imp. 2	Imp. 3	Imp. 4	Imp. 5	Imp. 6	Imp. 7	Imp. 8	Imp. 9	Imp. 10	Imp. 11	Imp. 12	Imp. 13	Imp. 14	Imp. 15	Imp. 16
0	1.153	1.148	1.156	1.139	1.149	1.143	1.145	1.145	1.148	1.149	1.145	1.153	1.141	1.147	1.148	1.150
5	1.156	1.150	1.155	1.136	1.152	1.145	1.142	1.158	1.151	1.160	1.135	1.143	1.143	1.155	1.144	1.132
10	1.134	1.143	1.144	1.147	1.148	1.147	1.140	1.143	1.145	1.150	1.151	1.144	1.146	1.150	1.140	1.143
15	1.132	1.140	1.143	1.146	1.160	1.145	1.147	1.137	1.152	1.162	1.123	1.125	1.159	1.143	1.142	1.137
20	1.078	1.078	1.081	1.081	1.086	1.083	1.078	1.083	1.079	1.082	1.085	1.084	1.082	1.082	1.079	1.082
25	1.068	1.060	1.065	1.068	1.073	1.064	1.066	1.072	1.071	1.064	1.071	1.064	1.062	1.070	1.061	1.070
30	1.024	1.059	1.044	1.043	1.041	1.031	1.067	1.067	1.044	1.059	1.047	1.047	1.035	1.070	1.044	1.073
35	1.008	1.039	1.061	1.052	1.051	1.058	1.036	1.039	1.067	1.059	1.061	1.033	1.021	1.016	1.046	1.067
40	1.035	1.035	1.035	1.036	1.037	1.036	1.036	1.035	1.036	1.036	1.036	1.036	1.036	1.035	1.036	1.036

BAC

Time (min)	Imp. 1	Imp. 2	Imp. 3	Imp. 4	Imp. 5	Imp. 6	Imp. 7	Imp. 8	Imp. 9	Imp. 10	Imp. 11	Imp. 12	Imp. 13	Imp. 14	Imp. 15	Imp. 16
0	1.145	1.152	1.148	1.151	1.148	1.150	1.150	1.152	1.150	1.148	1.152	1.150	1.151	1.156	1.153	1.153
5	1.117	1.120	1.118	1.118	1.116	1.117	1.118	1.117	1.117	1.116	1.118	1.117	1.117	1.117	1.118	1.120
10	1.091	1.089	1.093	1.087	1.091	1.090	1.086	1.092	1.093	1.093	1.091	1.086	1.087	1.091	1.086	1.083
15	1.075	1.083	1.081	1.080	1.083	1.078	1.084	1.084	1.082	1.079	1.079	1.081	1.079	1.081	1.081	1.081
20	1.076	1.078	1.078	1.078	1.081	1.077	1.076	1.078	1.078	1.079	1.076	1.079	1.073	1.077	1.076	1.078
25	1.079	1.058	1.072	1.085	1.081	1.080	1.087	1.076	1.086	1.071	1.078	1.085	1.079	1.088	1.086	1.076
30	1.081	1.065	1.087	1.062	1.069	1.072	1.075	1.089	1.068	1.081	1.071	1.062	1.077	1.067	1.082	1.068
35	1.068	1.068	1.073	1.060	1.069	1.069	1.069	1.064	1.063	1.059	1.060	1.074	1.074	1.065	1.074	1.062
40	1.064	1.065	1.065	1.065	1.064	1.065	1.065	1.065	1.065	1.065	1.065	1.064	1.064	1.064	1.065	1.065

Tween

Time (min)	Imp. 1	Imp. 2	Imp. 3	Imp. 4	Imp. 5	Imp. 6	Imp. 7	Imp. 8	Imp. 9	Imp. 10	Imp. 11	Imp. 12	Imp. 13	Imp. 14	Imp. 15	Imp. 16
0	1.142	1.143	1.144	1.150	1.149	1.156	1.155	1.144	1.138	1.150	1.138	1.158	1.145	1.142	1.146	1.152
5	1.146	1.145	1.146	1.148	1.148	1.145	1.147	1.144	1.146	1.145	1.145	1.146	1.145	1.148	1.149	1.144
10	1.135	1.133	1.141	1.139	1.142	1.126	1.133	1.141	1.142	1.136	1.132	1.148	1.149	1.137	1.138	1.142
15	1.142	1.137	1.132	1.137	1.139	1.136	1.140	1.135	1.143	1.133	1.136	1.133	1.128	1.136	1.131	1.134
20	1.130	1.132	1.140	1.131	1.116	1.127	1.128	1.127	1.135	1.129	1.130	1.132	1.125	1.128	1.131	1.139
25	1.114	1.144	1.134	1.124	1.120	1.132	1.114	1.125	1.123	1.119	1.134	1.138	1.117	1.121	1.136	1.133
30	1.121	1.147	1.127	1.131	1.097	1.123	1.136	1.123	1.149	1.124	1.112	1.095	1.119	1.115	1.117	1.123
35	1.098	1.082	1.123	1.117	1.113	1.101	1.082	1.097	1.109	1.131	1.110	1.097	1.099	1.108	1.081	1.116
40	1.098	1.096	1.097	1.098	1.096	1.095	1.094	1.097	1.093	1.103	1.092	1.098	1.098	1.097	1.097	1.101

b

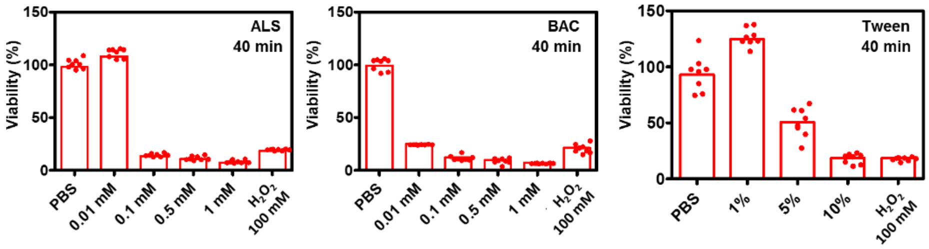

Supplementary Figure 23. Normalized impedance color maps (a) and cellular viabilities (b) of hDFB treated with ammonium lauryl sulfate (ALS), benzalkonium chloride (BAC), and Tween-60 (Tween) after 40 min. (n=8, mean)

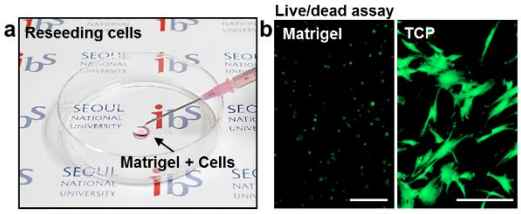

Supplementary Figure 24. Demonstration of reseeding the harvested hMSC. a, Photographic image of hMSC encapsulated within hydrogel (Matrigel, Corning) and reseeded onto the tissue culture plate (TCP). **b**, Fluorescence image showing the high viability of the harvested hMSC after encapsulation within hydrogel and the harvested hMSC reseeded onto TCP, as evaluated with the live/dead assay. Scale bars for Matrigel: 200 μm and for TCP: 50 μm.

Gene		Sequence
human GAPDH	Forward	TGC ACC ACC AAC TGC TTA GC
	Reverse	GGC ATG GAC TGT GGT CAT GAG
human caspase3	Forward	CTG GTT TTC GGT GGG TGT
	Reverse	CAG TGT TCT CCA TGG ATA CCT
mouse GAPDH	Forward	ATG TGT CCG TCG TGG ATC TGA
	Reverse	TGC CTG CTT CAC CAC CTT CT
mouse Myogenin	Forward	CTG ACC CTA CAG ACG CCC AC
	Reverse	TGT CCA CGA TGG ACG TAA GG
mouse MyoD	Forward	GGA AGA GTG CGG CTG TGT
	Reverse	CTG TTC TGT GTC GCT TAG GG

Supplementary Table 1. Primer sequences used in gene analysis.

Supplementary References

1. Wenger, R. H., Kurtcuoglu, V., Scholz, C. C., Marti, H. H., & Hoogewijs, D. Frequently asked questions in hypoxia research. *Hypoxia* **3**, 35 (2015)
2. Han, P. & Bartels, D. M. Temperature dependence of oxygen diffusion in H_2O and D_2O. *J. Phys. Chem.* **100**, 5597 (1996)
3. Jamnongwong, M., Loubiere, K., Dietrich, N., & Hebrard, G. Experimental study of oxygen diffusion coefficients in clean water containing salt, glucose or surfactant: consequences on the liquid-side mass transfer coefficients. *Chemical Engineering Journal* **165**, 758 (2010)
4. The Engineering Toolbox. https://www.engineeringtoolbox.com/oxygen-solubility-water-d_841.html
5. Li, W., Hu, Z., Chen, B., & Ni, G. Response of C2C12 myoblasts to hypoxia: the relative roles of glucose and oxygen in adaptive cellular metabolism. *BioMed Res. Int.* **2013**, 326346 (2013)
