# Supplementary material for: Large scale and integrated platform for digital mass culture of anchorage dependent cells
Source: Nat Commun. 2019 Oct 23;10:4824. doi: 10.1038/s41467-019-12777-3 (PMC6811641; doi:10.1038/s41467-019-12777-3)
Supplement: Supplementary file 3 — Reporting Summary [file 41467_2019_12777_MOESM3_ESM.pdf]

nature research

Corresponding author(s): Prof. Dae-Hyeong Kim and Prof. Byung Soo Kim

Last updated by author(s): Jun 16, 2019

Reporting Summary

Nature Research wishes to improve the reproducibility of the work that we publish. This form provides structure for consistency and transparency in reporting. For further information on Nature Research policies, see Authors & Referees and the Editorial Policy Checklist .

Statistics

For all statistical analyses, confirm that the following items are present in the figure legend, table legend, main text, or Methods section.

n/a	Confirmed	
☐	☒	The exact sample size (*n*) for each experimental group/condition, given as a discrete number and unit of measurement
☐	☒	A statement on whether measurements were taken from distinct samples or whether the same sample was measured repeatedly
☐	☒	The statistical test(s) used AND whether they are one- or two-sided<br>*Only common tests should be described solely by name; describe more complex techniques in the Methods section.*
☐	☒	A description of all covariates tested
☐	☒	A description of any assumptions or corrections, such as tests of normality and adjustment for multiple comparisons
☐	☒	A full description of the statistical parameters including central tendency (e.g. means) or other basic estimates (e.g. regression coefficient) AND variation (e.g. standard deviation) or associated estimates of uncertainty (e.g. confidence intervals)
☒	☐	For null hypothesis testing, the test statistic (e.g. *F*, *t*, *r*) with confidence intervals, effect sizes, degrees of freedom and *P* value noted<br>*Give P values as exact values whenever suitable.*
☒	☐	For Bayesian analysis, information on the choice of priors and Markov chain Monte Carlo settings
☒	☐	For hierarchical and complex designs, identification of the appropriate level for tests and full reporting of outcomes
☒	☐	Estimates of effect sizes (e.g. Cohen's *d*, Pearson's *r*), indicating how they were calculated

Our web collection on statistics for biologists contains articles on many of the points above.

Software and code

Policy information about availability of computer code

Data collection	Arduino 1.8.3; LabVIEW 2017
Data analysis	GraphPad Prism v5.03; OriginPro v8.0724

For manuscripts utilizing custom algorithms or software that are central to the research but not yet described in published literature, software must be made available to editors/reviewers. We strongly encourage code deposition in a community repository (e.g. GitHub). See the Nature Research guidelines for submitting code & software for further information.

Data

Policy information about availability of data

All manuscripts must include a data availability statement. This statement should provide the following information, where applicable:
- Accession codes, unique identifiers, or web links for publicly available datasets
- A list of figures that have associated raw data
- A description of any restrictions on data availability

The data that support the findings of this study are available from the corresponding author upon reasonable request.

Field-specific reporting

Please select the one below that is the best fit for your research. If you are not sure, read the appropriate sections before making your selection.

☒ Life sciences ☐ Behavioural & social sciences ☐ Ecological, evolutionary & environmental sciences

For a reference copy of the document with all sections, see nature.com/documents/nr-reporting-summary-flat.pdf

Life sciences study design

All studies must disclose on these points even when the disclosure is negative.

Sample size	We determined the sample size considering the requirement for statistical analysis.
Data exclusions	No data was excluded.
Replication	Biological replicate experiments were proceeded individually with fresh aliquots of cells.
Randomization	All the samples were chosen under randomization.
Blinding	The investigators were blinded to group allocation during data collection.

Reporting for specific materials, systems and methods

We require information from authors about some types of materials, experimental systems and methods used in many studies. Here, indicate whether each material, system or method listed is relevant to your study. If you are not sure if a list item applies to your research, read the appropriate section before selecting a response.

Materials & experimental systems

n/a	Involved in the study	
☐	☒	Antibodies
☐	☒	Eukaryotic cell lines
☒	☐	Palaeontology
☒	☐	Animals and other organisms
☒	☐	Human research participants
☒	☐	Clinical data

Methods

n/a	Involved in the study	
☒	☐	ChIP-seq
☒	☐	Flow cytometry
☒	☐	MRI-based neuroimaging

Antibodies

Antibodies used	myosin heavy-chain: mouse, abcam, ab683, 1:100<br>secondary antibody for myosin heavy-chain: donkey, abcam, ab150105, 1:100<br>connexin 43: rabbit, abcam, ab11370, 1:200<br>secondary antibody for connexin 43: goat, Jackson ImmunoResearch Laboratory, Inc. 111-095-144, 1:50
Validation	All commercial antibodies have been validated and published, with relevant information existing in the pertaining website.

Eukaryotic cell lines

Policy information about cell lines

Cell line source(s)	C2C12,CRL-1772,ATCC; hDFB, PCS-201-012, ATCC; HL-1, SCC065,Merck; hMSC, PT-2501,Lonza.
Authentication	C2C12 and hDFB purchased from ATCC within 5 years, hMSC purchased from Lonza within 4 years, and HL-1 purchased from Merck within 2 years were used in this study. Cells were also authenticated by the suppliers.
Mycoplasma contamination	Confirm that all cell lines tested negative for mycoplasma contamination
Commonly misidentified lines<br>(See ICLAC register)	No commonly misidentified cell lines were used in this study
